# Characterization of Chitosan Hydrogels Obtained through Phenol and Tripolyphosphate Anionic Crosslinking

**DOI:** 10.3390/polym16091274

**Published:** 2024-05-02

**Authors:** Mitsuyuki Hidaka, Masaru Kojima, Shinji Sakai, Cédric Delattre

**Affiliations:** 1 Division of Chemical Engineering, Department of Materials Engineering Science, Graduate School of Engineering Science, Osaka University, 1-3 Machikaneyama-cho, Toyonaka, Osaka 560-8531, Japan; hidakauo@cheng.es.osaka-u.ac.jp (M.H.); kojima@cheng.es.osaka-u.ac.jp (M.K.); sakai@cheng.es.osaka-u.ac.jp (S.S.); 2 Université Clermont Auvergne, Clermont Auvergne INP, CNRS, Institut Pascal, 63000 Clermont-Ferrand, France; 3 Institute Universitaire de France (IUF), 1 rue Descartes, 75005 Paris, France

**Keywords:** chitosan, hydrogel, phenol group, tripolyphosphate

## Abstract

Chitosan is a deacetylated polymer of chitin that is extracted mainly from the exoskeleton of crustaceans and is the second-most abundant polymer in nature. Chitosan hydrogels are preferred for a variety of applications in bio-related fields due to their functional properties, such as antimicrobial activity and wound healing effects; however, the existing hydrogelation methods require toxic reagents and exhibit slow gelation times, which limit their application in biological fields. Therefore, a mild and rapid gelation method is necessary. We previously demonstrated that the visible light-induced gelation of chitosan obtained through phenol crosslinking (ChPh) is a rapid gelation method. To further advance this method (<10 s), we propose a dual-crosslinked chitosan hydrogel obtained by crosslinking phenol groups and crosslinking sodium tripolyphosphate (TPP) and the amino groups of chitosan. The chitosan hydrogel was prepared by immersing the ChPh hydrogel in a TPP solution after phenol crosslinking via exposure to visible light. The physicochemical properties of the dual-crosslinked hydrogels, including Young’s moduli and water retentions, were subsequently investigated. Young’s moduli of the dual-crosslinked hydrogels were 20 times higher than those of the hydrogels without TPP ion crosslinking. The stiffness could be manipulated by varying the immersion time, and the water retention properties of the ChPh hydrogel were improved by TPP crosslinking. Ion crosslinking could be reversed using an iron chloride solution. This method facilitates chitosan hydrogel use for various applications, particularly tissue engineering and drug delivery.

## 1. Introduction

Hydrogels are three-dimensional networks of polymer chains that are filled with water. Hydrogels are soft and biocompatible owing to their abundance in water. In particular, polysaccharide-based hydrogels have attracted considerable attention for various bio-related applications, such as tissue engineering and biosensors [1,2,3,4]. Unlike synthetic polymers, polysaccharides can be extracted from renewable sources, are inexpensive [5,6], and have excellent biofunctional and physicochemical properties [5,7,8,9].

Among them, chitosan hydrogels exhibit excellent physicochemical properties for bio-related applications [10,11,12]. Chitosan is a deacetylated polymer of chitin, the second-most abundant polysaccharide in nature [11,13]. Chitosan/chitin is extracted mainly from the exoskeleton of crustaceans, such as crabs and shrimp. Insects and fungi are also sources of chitosan [14,15]. Chitosan hydrogels exhibit desirable properties for various bio-related applications, including drug delivery and tissue engineering, owing to their cationic, antimicrobial, and antioxidant properties [13,16,17,18].

Chitosan hydrogels have been obtained using various methods, including physical crosslinking, which is a widely used method [19,20,21,22]. For example, chitosan is soluble in acidic aqueous solutions, and a chitosan hydrogel is obtained with an increase in pH due to its solubility change [23]. Chemical crosslinking has also been used [24]. Genipin, a natural crosslinker from *Genipa americana*, forms covalent bonds between chitosan polymer chains and is prepared by mixing chitosan aqueous solutions with genipin [25,26]. However, these gelation processes are slow (more than 1 h), which could be the rate-determining step in the overall process of chitosan application [25]. To obtain the homogeneous hydrogel, mixing the crosslinker with the polymer solution is required; therefore, the process is time-consuming. In addition, crosslinkers that are toxic and harsh in the presence of animal cells [23], such as NaOH, are required, which limits the properties of the hydrogels, as well as the crosslinker concentration. Therefore, rapid and mild gelation is desirable to widen their applications.

To overcome these limitations, we developed a phenolic derivative of chitosan (ChPh) [27]. The phenol groups introduced to chitosan were crosslinked with sodium persulfate (SPS) and Ru(bpy)_3_ through visible light exposure, resulting in the formation of a chitosan hydrogel. In this reaction, an electron of Ru(bpy)_3_ is excited by light irradiation, promoting a SPS radical that promotes crosslinking between the phenol groups introduced to the polymers. The light-induced gelation process enables rapid and homogeneous gelation, unlike the other crosslinking processes described above. The gelation time of this method was fast (<1 min), which is suitable for fabricating various 3D structures [28,29]. However, the hydrogel exhibited low stiffness [30,31], and a hydrogel with a higher stiffness is required for stable and long-term use.

**Figure 1:**
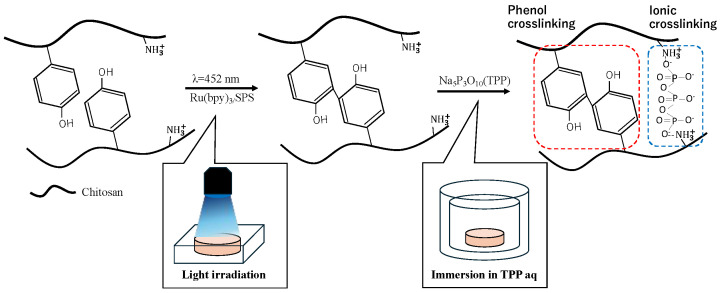
Dual-crosslinked chitosan hydrogel obtained by phenol and TPP crosslinking.

In this study, we propose a dual-crosslinked chitosan hydrogel obtained by phenol and sodium tripolyphosphate (TPP) crosslinking (Figure 1). TPP crosslinking is induced by the ionic interactions between the tripolyphosphate anion and the protonated amino groups of chitosan [32,33]. We evaluated the synergistic effect of phenol and ionic crosslinking on several properties, including stiffness and swelling behavior, for understanding the hydrogel stability in physicochemical and physiological environments. These results will facilitate the use of chitosan hydrogels in various applications.

## 2. Materials and Methods

### 2.1. Materials

Chitosan (high molecular weight chitosan from crab, 500 kDa, deacetylation degree 75%, purity ≤ 100%), sodium persulfate (purity ≥ 98%), Ru(bpy)_3_·Cl_2_·6H_2_O (purity ≥ 99.95%), 1-ethyl-3-(3-dimethylaminopropyl)carbodi-imide hydrochloride (EDC·HCl, purity ≥ 97%), 3-(4-hydroxyphenyl) propionic acid (HPP, purity ≥ 96%), N,N,N′,N′-Tetramethylethylenediamine (TEMED, purity = 98%), and TPP (purity = 98%) were purchased from Sigma Aldrich (St. Louis, MO, USA). *Escherichia coli* (OP50) was cultured in a Luria Broth (LB) medium containing 0.5 wt% NaCl, 1 wt% Bacto tryptone (Becton Dickinson and Company, Franklin Lakes, NJ, USA), and Bacto yeast extract (Becton Dickinson and Company, Franklin Lakes, NJ, USA).

### 2.2. ChPh Synthesis

The phenol group was introduced with chitosan, based on a previously reported method [29,34,35]. Briefly, chitosan was dissolved in 20 mM HCl at a concentration of 2.0 wt%. TEMED was added to the solution at 2.0 wt%, and the pH was adjusted to 5 using NaOH and HCl. Thereafter, EDC·HCl, lactobionic acid, and HPP were added to the solution at 1, 0.04, and 1.5 wt%, respectively. After stirring for 20 h, the reaction was stopped by adding excess acetone. The precipitate was rinsed several times with an 80 wt% EtOH aqueous solution. The solution was dehydrated with pure ethanol and dried in the oven at 45 °C overnight. The modification of phenol groups to chitosan was confirmed (Figure S1) by UV–Vis spectrometry, and the amount was 7.5 × 10^−6^ mol/g–ChPh, as calculated from the absorbance of phenol groups according to the method described in the literature [36].

### 2.3. Comparison of Chitosan Hydrogels Obtained Using Different Crosslinking Methods

The chitosan hydrogels were prepared using the following four gelation methods: (a) 125 µL of ChPh aqueous solution (2.0 wt%) was poured into the circle mold with a 12 mm diameter and 5 mm depth, and 1.5 wt% TPP aqueous solution was added to the ChPh aqueous solution; (b) 125 µL of ChPh aqueous solution containing 4 mM of SPS and 1 mM of Ru(bpy)_3_ was poured into the well and exposed to visible light (λ = 452, 8.0 W/m^2^) for 20 min; (c) 125 µL of ChPh aqueous solution containing 4 mM of SPS and 1 mM of Ru(bpy)_3_ was poured into the well, and 1.5 wt% TPP aqueous solution was added to the ChPh aqueous solution; after 5 min, the sample was exposed to visible light for 20 min; and (d) 125 µL of ChPh aqueous solution containing 4 mM of SPS and 1 mM of Ru(bpy)_3_ was poured into the well and exposed to visible light for 20 min. These reagent concentrations and light irradiation conditions were based on previous studies [29,37]. Subsequently, 1.5 wt% TPP aqueous solution was added to the ChPh aqueous solution.

### 2.4. TPP Phenol-Crosslinked Hydrogel Preparation

Approximately 125 µL of sample aqueous solution (2.0 wt% of ChPh), 1–4 mM of SPS, and 1 mM of Ru(bpy)_3_ were poured into the mold. The sample was then exposed to the visible light (λ = 452 nm, 8.0 W/m^2^) for phenol crosslinking for 20 min. The sample was removed from the mold with a spatula and immersed in 30 mL of 1.5 wt% TPP aqueous solution for TPP crosslinking for 1, 5, and 10 min (denoted as ChPh–TPP1, ChPh–TPP5, and ChPh–TPP10, respectively). These samples were used for the series of experiments described below.

### 2.5. Fourier-Transform Infrared Spectroscopy

Fourier-transform infrared (FTIR) spectra of ChPh, ChPh–TPP1, ChPh–TPP5, and ChPh–TPP10 were measured by using a FT/IR-4100 instrument (JASCO, Tokyo, Japan). Each hydrogel sample was dried and milled, and a KBr tablet containing each sample (KBr: sample = 100:1) was prepared for the measurement. Thirty scans at a resolution of 4 cm^−1^ were conducted.

### 2.6. Young’s Modulus

The mechanical properties of the ChPh–TPP1, ChPh–TPP5, and ChPh–TPP10 hydrogels were determined by measuring the repulsive forces toward compression (6 mm/min) using a tabletop materials tester (EZ-test, Shimadzu, Kyoto, Japan). The SPS and Ru(bpy)_3_ concentrations were 1–4 mM and 1 mM, respectively. Young’s moduli were calculated using the stress data when 1–10% strain was applied to the sample. The Young’s modulus of each sample was compared with ChPh without ionic crosslinking. Three samples from each group of hydrogels were evaluated, and the average value was recorded.

### 2.7. Degree of Swelling

The degree of swelling was measured according to a previously described method [38]. ChPh–TPP1, ChPh–TPP5, and ChPh–TPP10 hydrogels were prepared at 1–4 mM SPS and 1 mM Ru(bpy)_3_. The sample hydrogel was immersed in phosphate-buffered saline (PBS, pH 7.4) at 20 °C for 5 h. After that, the residual water on the surface of the hydrogel was removed with a paper towel. The wet weight (*W_w_*) was measured, and the weight ratio of the hydrogel against the initial hydrogel weight was calculated using the following equation:

(1) $$\mathrm{Swelling}\;\mathrm{degree}\;[-]=\frac{W_{w}}{W_{0}}$$

where *W*_0_ is the initial weight, and *W_w_* is the wet weight of the sample. When this value is higher than 1, it shows the swelling of the hydrogel. Three samples from each group of hydrogels were evaluated, and the average value was recorded.

### 2.8. Water Retention

The water retention was measured using a previously described method [38]. The hydrogel sample was placed in a 24-well plate and incubated at 20 °C for 5 h. SPS and Ru(bpy)_3_ concentrations of 4.0 mM and 1 mM, respectively, were used. The weights of the hydrogels were measured, and water retention was evaluated using the following equation:

(2) $$\mathrm{Water}\;\mathrm{retention}\;[\%]=\frac{W_{t}}{W_{0}}$$

where *W*_0_ is the initial weight, and *W_t_* is the weight of the samples after *t* h. Five samples from each group of hydrogels were evaluated, and the average value was recorded.

### 2.9. Antimicrobial Activity

The antimicrobial activity of each sample was evaluated using the Gram-negative bacteria *E. coli.* The bacteria were cultured in a (LB) medium. A sample hydrogel (ChPh, ChPh–TPP1, ChPh–TPP5, and ChPh–TPP10 at 4 mM SPS) was immersed in 2 mL of the LB medium containing the bacteria at 10^8^ CFU/mL at 37 °C overnight in a shaking incubator. The OD_600_ of each medium was measured, and the CFU value was calculated based on a previously described method [39]. Three samples from each group of hydrogels were evaluated, and the average value was recorded.

### 2.10. Removal of Ionic Crosslinking

It has been reported that chitosan hydrogels crosslinked with TPP chelate metal ions [40,41]. To test whether ionic crosslinking could be removed, ChPh–TPP5 at 4 mM SPS was immersed in 1 wt% FeCl_3_ aqueous solution for 15 min. After immersion, the residual water on the surface of the hydrogels was removed with a paper towel and they were observed to check for shape changes.

### 2.11. Statistical Analysis

Statistical analysis was performed using the Student’s *t*-test. A *p*-value of < 0.05 was considered statistically significant. A spreadsheet software, Excel (ver16.79, Microsoft, Redmond, WA, USA), was used for the analysis.

## 3. Results and Discussion

### 3.1. Comparison of Chitosan Hydrogels Obtained Using Different Crosslinking Methods

**Figure 2:**
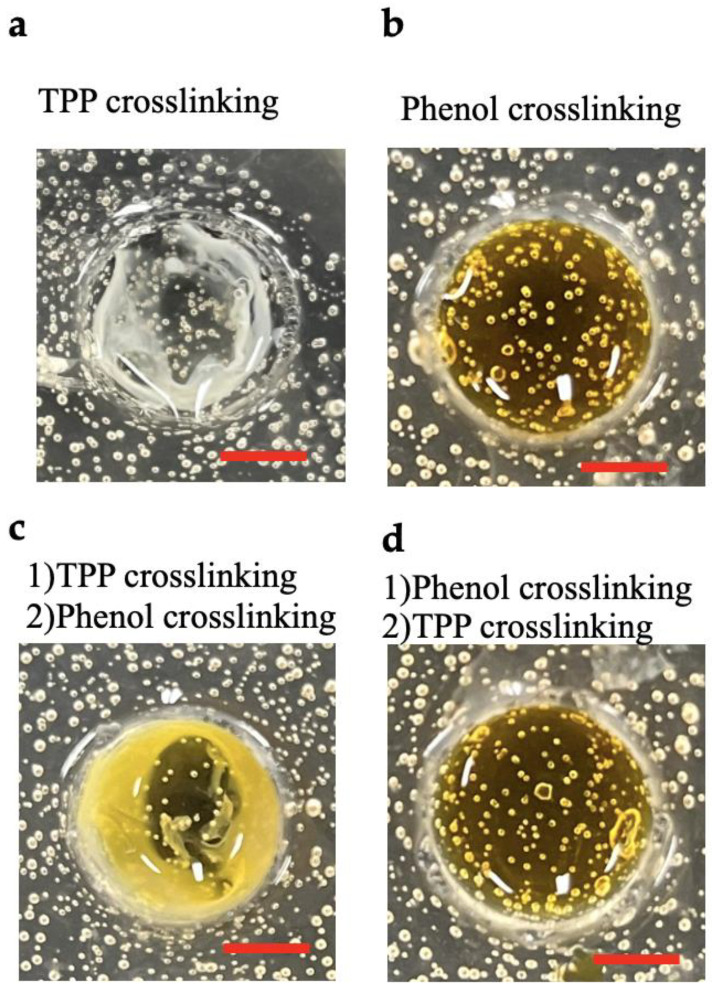
Comparison of chitosan hydrogel obtained using four different crosslinking methods: (**a**) sodium TPP crosslinking for 5 min (left top), (**b**) phenol crosslinking and exposure to visible light for 20 min (right top), (**c**) phenol crosslinking after TPP crosslinking for 5 min (left bottom), and (**d**) TPP crosslinking for 5 min after phenol crosslinking with exposure to visible light for 20 min (right bottom). Scale bar = 5 mm.

First, we compared the stability of chitosan hydrogels obtained using four different crosslinking methods. In the first crosslinking method, 125 µL of ChPh aqueous solution (2.0 wt%) was poured into a circle mold with a 12 mm diameter, and 1.5 wt% TPP aqueous solution was added to the ChPh aqueous solution. We observed shrinkage of the sample 5 min after adding the TPP solution (Figure 2a). In the second method, 125 µL of ChPh aqueous solution with 4 mM of SPS and 1 mM of Ru(bpy)_3_ was poured into the mold and exposed to visible light (λ = 452, 8.0 W/m^2^) for 20 min (Figure 2b). The sample was stable, and no shrinkage was observed. In the third method, 125 µL of ChPh aqueous solution containing 4 mM of SPS and 1 mM of Ru(bpy)_3_ was poured into the mold, and 1.5 wt% TPP aqueous solution was added to the ChPh aqueous solution. After 5 min, the sample was exposed to visible light for 20 min. Shrinkage was observed, similar to that observed in the first sample (Figure 2c). In the fourth method, 125 µL of ChPh aqueous solution containing 4 mM of SPS and 1 mM of Ru(bpy)_3_ was poured into the mold and exposed to visible light for 20 min. Subsequently, 1.5 wt% TPP aqueous solution was added to the ChPh aqueous solution. After 5 min, no shrinking was observed (Figure 2d). For the successful application of chitosan hydrogels, it is desirable for the hydrogel to retain its shape for the fabrication and stabilization of the structure [42,43]. Our findings suggest that the chitosan hydrogel obtained by TPP crosslinking after phenol crosslinking is more versatile than that obtained by TPP and phenol crosslinking after TPP crosslinking, as it retains its shape.

### 3.2. FTIR Spectroscopy

**Figure 3:**
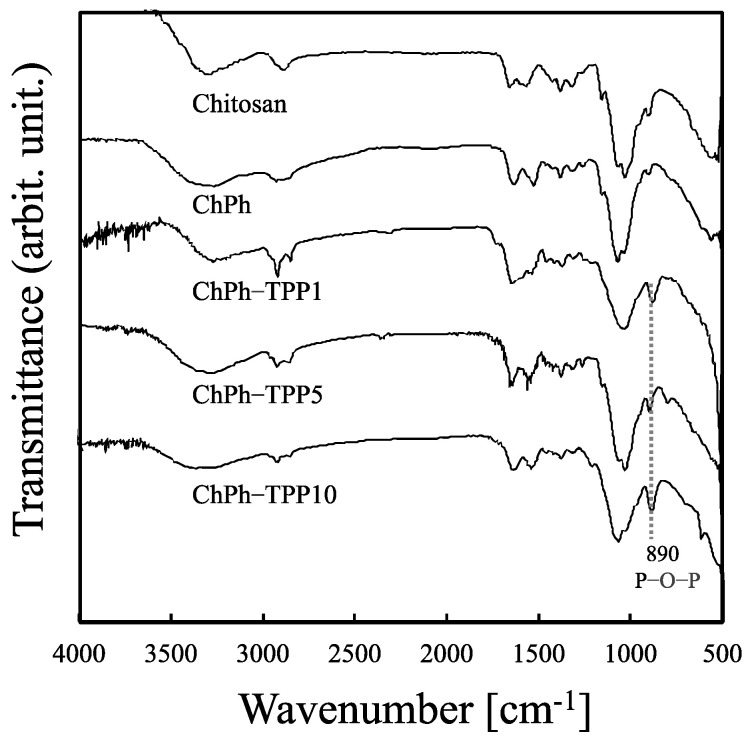
FTIR spectra of chitosan, ChPh, ChPh–TPP1, ChPh–TPP5, and ChPh–TPP10.

We showed the crosslinking order was important to obtain a stable hydrogel, as described above. FTIR spectroscopy was used to understand the chemical structures of the dual-crosslinked chitosan hydrogels obtained by TPP crosslinking after light-induced crosslinking (ChPh, ChPh–TPP1, ChPh–TPP5, and ChPh–TPP10). In ChPh, a broad peak was observed at around 3300 cm^−1^, which is attributed to –NH_2_ and –OH groups streching (Figure 3). The charcteristic bands at around 2900 cm^−1^ are attributed to C–H symmetry and asymmetry streching. The band at around 1645 cm^−1^ is attributed to C=O stretching of amide I. The band at around 1580 cm^−1^ is attributed to N–H bending of the primary amine. The characteristic band at around 1060 cm^−1^ is attributed to C–O streching. These resuts corresponded well to the previous literature [44,45]. In ChPh–TPP1, ChPh–TPP5, and ChPh–TPP10, sharp peaks were observed at 890 cm^−1^ attributed to P–O–P stretching, which shows the presence of TPP inside the hydrogels [46,47]. However, any other significant difference was not observed among the samples.

### 3.3. Young’s Modulus

**Figure 4:**
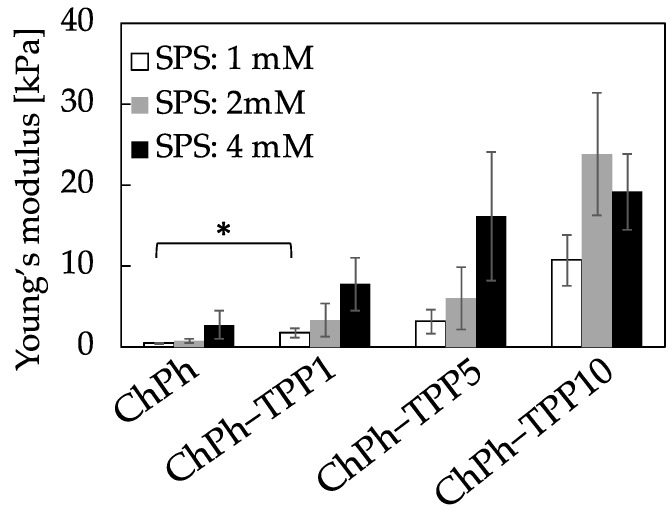
Effect of TPP immersion time on the Young’s modulus. The ChPh aqueous solutions containing SPS and Ru(bpy)_3_ were gelated by exposure to visible light and were subsequently immersed in TPP solution for 1, 5, and 10 min (denoted as ChPh–TPP1, ChPh–TPP5, and ChPh–TPP10, respectively). Data: ±mean S.D. (*n* = 3–5), * *p* < 0.05.

To understand the effect of TPP immersion time on the mechanical properties of the dual-crosslinked chitosan hydrogel, the Young’s modulus was measured. The Young’s modulus increased with an increase in the TPP immersion time (Figure 4). For example, the Young’s modulus of ChPh–TPP1 at 1 mM of SPS was 10.7 ± 3.1 kPa, while that of ChPh at the same SPS concentration without TPP crosslinking was 0.53 ± 0.06 kPa (*p* < 0.05). The maximum Young’s modulus was 23.7 ± 7.6 kPa at ChPh–TPP10 at 2 mM of SPS. These results suggest that the Young’s modulus of ChPh–TPP was approximately 20 times higher than that of the hydrogel without TPP crosslinking. Reportedly, hydrogels obtained through phenol crosslinking are biocompatible; however, their stiffness is weak and fragile (<10 kPa) [48,49], thereby limiting their application in biological fields. The mechanical properties are an important factor in the rigidity of the hydrogel and play an important role in cell growth and proliferation [50,51]. We demonstrated that the mechanical properties of ChPh improved after TPP crosslinking and could be manipulated by changing the immersion time in the TPP aqueous solution.

### 3.4. Degree of Swelling

**Figure 5:**
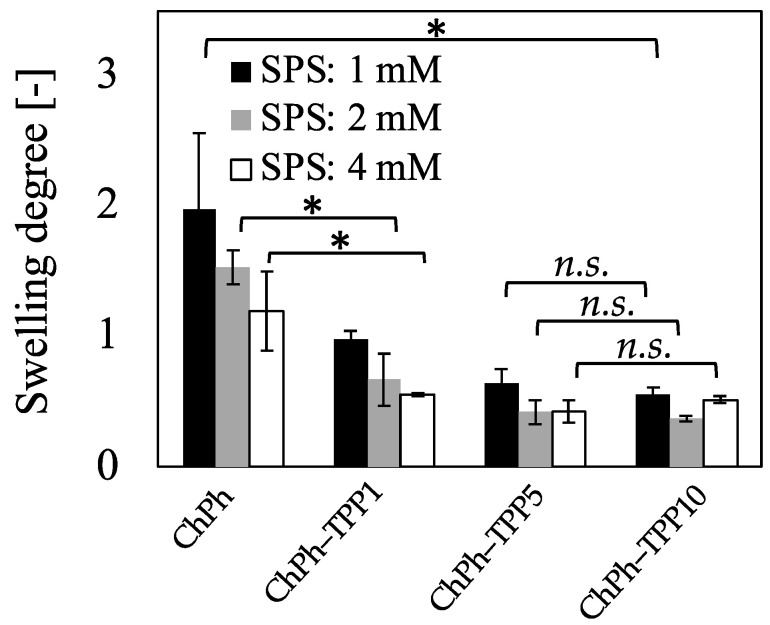
Effect of TPP immersion time on the swelling degree after 5 h of immersion in PBS. Data: ±mean S.D. (*n* = 3), * *p* < 0.05, *n.s.* > 0.1.

We investigated the degree of swelling of the ChPh–TPP hydrogel to understand its swelling behavior at room temperature (Figure 5). Each sample was immersed in PBS. The equilibrium of the swelling degree was confirmed at 5 h (Figure S2). The weights of the samples are listed in Table S1. We found that the dual-crosslinked chitosan hydrogel shrunk in the solution, whereas the hydrogel without TPP crosslinking expanded. For example, the weight ratio of ChPh was 197 ± 59% at 1 mM of SPS, whereas that of ChPh–TPP10 was 55 ± 5% at the same SPS concentration (*p* < 0.01). Similar shrinkage effects were observed for ChPh–TPP1 at 2 and 4 mM SPS (*p* < 0.01). There was no significant difference between the degree of swelling of ChPh–TPP5 and ChPh–TPP10 at any SPS concentration (*p* > 0.1). These findings suggest that TPP crosslinking occurred inside the ChPh hydrogel even after its removal from the TPP solution, causing the hydrogel to shrink until the interaction between TPP and the amino group of chitosan reached equilibrium. This was also likely caused by the hydrophobicity of the phenol groups, as it has been reported that phenol groups are hydrophobic [52,53]. However, the amino groups of chitosan are hydrophilic because they are protonated and interact with water. This hydrophilic interaction was reduced by ionic crosslinking between the amino group and TPP anion, which enhanced the hydrophobic interaction of the phenol group introduced with chitosan. Hydrophobic materials have been widely used as drug carriers for cancer because of their hydrophobicity, which is a result of their molecular structures and functional groups [54,55]. These findings suggest that this dual-crosslinked hydrogel has the potential for use as a drug carrier owing to its hydrophobicity.

### 3.5. Water Retention

**Figure 6:**
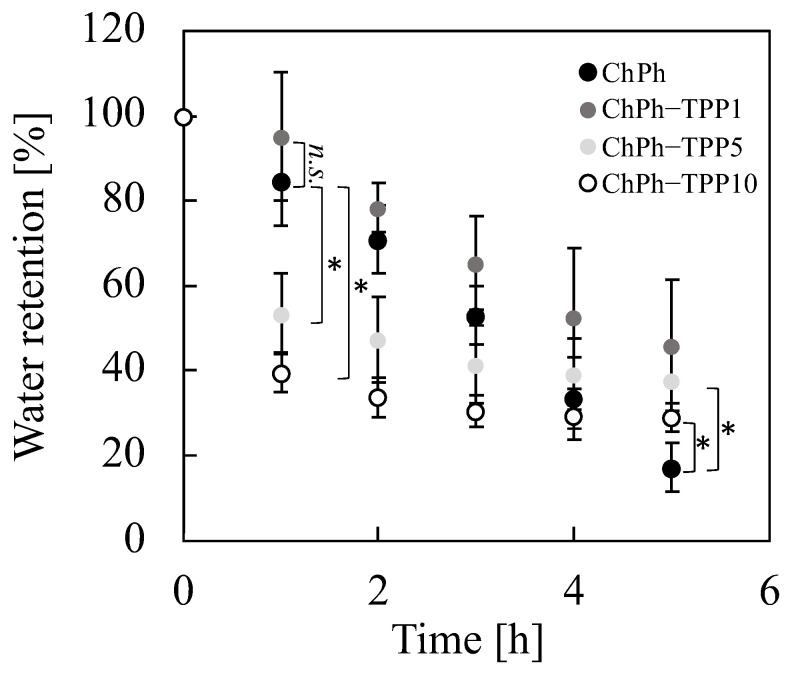
Effect of TPP immersion time on water retention. Data: ±mean S.D. (*n* = 5), * *p* < 0.05.

In addition to the swelling degree measurements, water retention was measured to further understand the properties of the ChPh–TPP hydrogel (Figure 6). ChPh hydrogels with different TPP immersion times were prepared using a 4 mM SPS and 1 mM Ru(bpy)_3_ solution. Each sample was placed in a 24-well plate at 20 °C, and the weight of the hydrogel was measured hourly. The weights of the samples are listed in Table S2. The ChPh sample became dry in 5 h. The water retention of ChPh was higher than those of ChPh–TPP5 and ChPh–TPP10 (*p* < 0.01, Figure 5) after 1 h. No significant differences were observed between ChPh and ChPh–TPP1 (*p* > 0.1). Our results suggest that ChPh–TPP5 and Ch–TPP10 shrank owing to internal TPP crosslinking, even after being removed from the TPP solution, as described in Section 2.3. In contrast, after 5 h, the ChPh hydrogel without TPP crosslinking exhibited the lowest water retention, while ChPh–TPP1 exhibited the highest value. In addition, the decrease in the water retention of ChPh–TPP5 and ChPh–TPP10 was steady and slow, and the final water retention values of these samples were higher than those of ChPh (*p* < 0.01) after 5 h. In general, the density of the crosslinking network strongly affects the swelling and shrinking of the hydrogel. At a high crosslinking density, the diffusion of the solvent inside the hydrogel into the atmosphere is disturbed owing to the presence of resistant paths [45,46]. Hence, it was suggested that the paths inside ChPh–TPP5 and ChPh–TPP10 improved the water retention with an increase in the TPP immersion time, although they still shrunk. The water retention of hydrogels is an important property for the long-term use and controlled release of nutrients and drugs in bio-related applications [56,57]. Our results revealed that the immersion of the ChPh hydrogel in TPP solution improved the water retention.

### 3.6. Antimicrobial Activity

**Figure 7:**
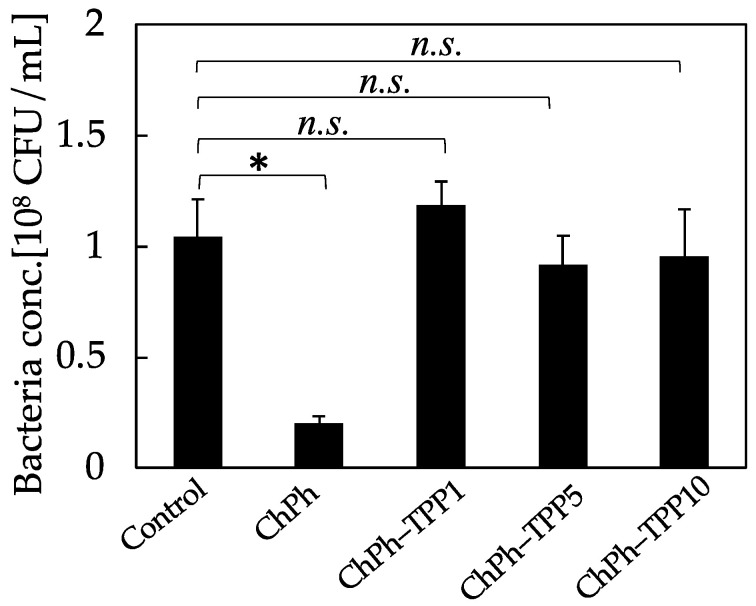
Effect of TPP immersion time on antimicrobial activity. Data: ±mean S.D. (*n* = 3), * *p* < 0.05, *n.s.* > 0.1.

The antimicrobial activity of each sample was tested. As a control sample, *E. coli* was cultured overnight in a LB medium for one night. In addition, the ChPh and ChPh–TPP hydrogels were placed in a LB medium containing *E. coli* and were cultured for one night. Subsequently, the OD_600_ of each medium was measured, and the colony-forming units (CFUs) were calculated based on the literature [39]. The CFU value of ChPh was lower than that of the control (*p* < 0.05; Figure 7). This result corresponded to that of our previous study [29]; ChPh hydrogel has antimicrobial activity. In contrast, the chitosan hydrogels obtained by TPP and phenol crosslinking exhibited no significant difference in CFU values compared to the control. The antimicrobial activity of chitosan originates from its cationic properties owing to its protonated amino groups [13,18,58]. Upon the addition of TPP, the tripolyphosphate anion interacts with the amino cation. We believe that this interaction neutralizes the cationic properties of chitosan. Cationic properties are important for drug carriers and cell attachment. Although this cationic property was not controlled in this study, it may be possible to control it by changing the immersion time and TPP concentration [32].

### 3.7. Removal of Ionic Crosslinking

**Figure 8:**
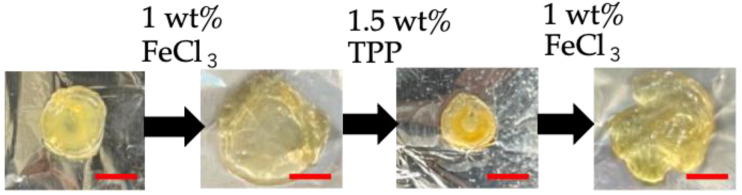
Removal and reversibility of TPP crosslinking using FeCl_3_ (scale bar = 5 mm).

To investigate the reversibility of TPP ionic gelation, the ChPh–TPP5 hydrogel was immersed in a FeCl_3_ solution. The hydrogels swelled after 15 min and shrank again when immersed in TPP solution (Figure 8). These results indicate that TPP ionic crosslinking is reversible. Metal adsorption by chitosan for metal removal has been widely reported [40,59]. Chelates are formed between iron ions and amino and hydroxy groups of chitosan. This suggests that the TPP anions interacting with the amino groups of chitosan are replaced by iron ions. Chitosan hydrogels obtained via physical or ionic crosslinking have been used for this purpose. However, these hydrogels are generally physicochemically unstable, because their stability critically depends on the pH environment around the hydrogel [60,61]. In contrast, phenol crosslinking involves covalent bonds, facilitating physicochemical stability. In addition, the phenol group can be introduced on stable substrates, such as graphene and glass [62,63]. Hence, the ChPh–TPP hydrogel could be fixed on such a substrate and used as a filler material for metal absorption, as it maintains the hydrogel state owing to phenol crosslinking, even after cleavage of the TPP anion and absorption of the metal iron. In addition, the state of hydrogel-absorbed iron could be visually observed. This property may enable the sensing of iron in aqueous solutions based on its appearance. However, this is just a qualitative analysis, and further investigation will be required for its practical application.

## 4. Conclusions

In this study, we propose a chitosan hydrogel with covalent bonds between phenol groups and ion bonds between the TPP anions and amino cations of chitosan. We demonstrated that the Young’s modulus of the dual-crosslinked hydrogel was approximately 20 times higher than that of the ChPh hydrogel without TPP, and the mechanical properties of the gel could be manipulated by changing the immersion time. The result indicates that this dual-crosslinking method expands the application potential of the phenol-crosslinked chitosan hydrogel. In addition, the degree of swelling and water retention of the hydrogels were evaluated. Although the swelling of the ChPh hydrogels with TPP crosslinking decreased, their water retention properties improved with an increase in the TPP immersion time. Furthermore, ion crosslinking could be reversed by immersing the hydrogel in an iron chloride solution. Our findings suggest that this material has the potential to be used for various applications, including as a drug carrier and filter material for metal. However, further investigation would be needed, such as a drug load/release amount test, pore size measurement, and spectroscopic measurements.

## Data Availability

The raw data supporting the conclusions of this article will be made available by the authors on request.

## Supplementary Materials

The following supporting information can be downloaded at https://www.mdpi.com/article/10.3390/polym16091274/s1: Figure S1: UV–Vis spectrum of 0.1 wt% chitosan (Ch) and ChPh solution (solvent: 20 mM HCl); Figure S2: Time–course measurement of the swelling degree of ChPh, ChPh–TPP1, ChPh–TPP5, and ChPh–TPP10 (*n* = 3, Data: ±S.D.); Table S1: Weight (mg) comparison of ChPh, ChPh–TPP1, ChPh–TPP5, and ChPh–TPP10 after 5 h of immersion in PBS, *n* = 3. Data: ±S.D.; Table S2: Weight (mg) comparison of ChPh, ChPh–TPP1, ChPh–TPP5, and ChPh–TPP10 after 5 h of incubation at 20 °C, *n* = 5. Data: ±S.D.
