# Supplementary material for: Characterization of Chitosan Hydrogels Obtained through Phenol and Tripolyphosphate Anionic Crosslinking
Source: Polymers (Basel). 2024 May 2;16(9):1274. doi: 10.3390/polym16091274 (PMC11085344; doi:10.3390/polym16091274)
Supplement: Supplementary file 1 [file polymers-16-01274-s001.zip › polymers-2939262-supplementary.pdf]

polymers

MDPI

Supporting information

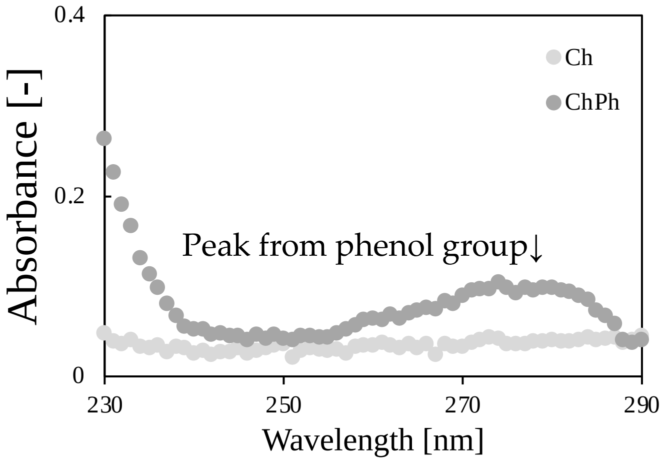

Figure S1. UV-vis spectrum of 0.1 wt% chitosan (Ch) and ChPh solution (solvent: 20 mM HCl)

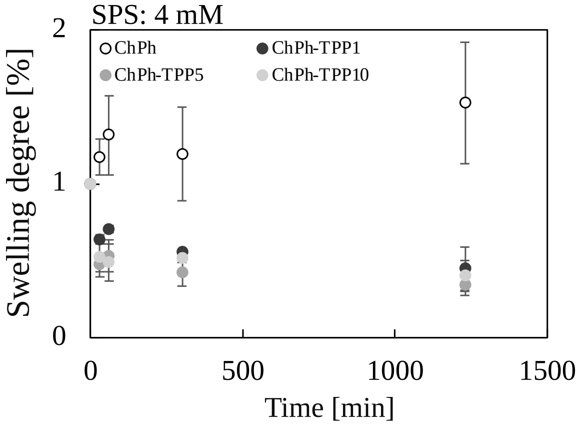

Figure S2. Time-course measurement of swelling degree of ChPh, ChPh-TPP1, 5, and 10 (n=3, Data:±S.D.)

Table S1. Weight (mg) comparison of ChPh, ChPh-TPP1,5, and 10 after 5 h of immersion in PBS , n=3, Data:±S.D.

SPS 1 mM	ChPh	ChPh-TPP1	ChPh-TPP5	ChPh-TPP10
0h	126±40	118±10	90±16	84±19
5h	263±140	116±14	59±20	45±6

SPS 2 mM	ChPh	ChPh-TPP1	ChPh-TPP5	ChPh-TPP10
0 h	154±22	131±34	124±11	104±10
5 h	238±46	92±53	52±10	38±4

SPS 4 mM	ChPh	ChPh-TPP1	ChPh-TPP5	ChPh-TPP10
0 h	170±20	114±18	81±18	72±5
5 h	201±51	63±9	33±3	37±4

Table S2. Weight (mg) comparison of ChPh, ChPh-TPP1,5, and 10 after 5 h of incubation at 20 ˚C, n=5, Data:±S.D.

	ChPh	ChPh-TPP1	ChPh-TPP5	ChPh-TPP10
0 h	147±24	132±12	150±28	107±27
5 h	26±11	61±23	58±20	32±11
